# Associations between systemic inflammation, mycobacterial loads in sputum and radiological improvement after treatment initiation in pulmonary TB patients from Brazil: a prospective cohort study

**DOI:** 10.1186/s12879-016-1736-3

**Published:** 2016-08-05

**Authors:** Eliene D. D. Mesquita, Leonardo Gil-Santana, Daniela Ramalho, Elise Tonomura, Elisangela C. Silva, Martha M. Oliveira, Bruno B. Andrade, Afrânio Kritski, for the Rede-TB Study group

**Affiliations:** 1 Ary Parreira Institute, State Secretary of Health of Rio de Janeiro, Rio de Janeiro, Brazil; 2 Unidade de Medicina Investigativa, Laboratório Integrado de Microbiologia e Imunorregulação (LIMI), Centro de Pesquisas Gonçalo Moniz, Fundação Oswaldo Cruz, Salvador, Brazil; 3 Multinational Organization Network Sponsoring Translational and Epidemiological Research (MONSTER) Initiative, Fundação José Silveira, Salvador, Brazil; 4 School of Medicine, Faculdade de Tecnologia e Ciências, Salvador, Brazil; 5 Tuberculosis Academic Program, School of Medicine, Federal University of Rio de Janeiro, Rio de Janeiro, Brazil; 6 Radiology Department, School of Medicine, Federal University of Rio de Janeiro, Rio de Janeiro, Brazil; 7 Recognize the Biology Laboratory, Center of Bioscience and Biotechnology, State University of North Fluminense Darcy Ribeiro, Rio de Janeiro, Brazil; 8 Development Center for Technology on Health, Fundação Oswaldo Cruz, Rio de Janeiro, Brazil

**Keywords:** Tuberculosis, Inflammation, C-reactive protein, Biomarker, Erythrocyte sedimentation rate, Acid-fast bacilli, Anti-tuberculous treatment, Radiographic evaluation

## Abstract

**Background:**

*Mycobacterium tuberculosis* infection is known to cause inflammation and lung tissue damage in high-risk populations. Nevertheless, direct associations between mycobacterial loads, systemic inflammation and pulmonary lesions upon treatment initiation have not been fully characterized. In the present exploratory study, we prospectively depict the immune profile, microbial clearance and evolution of radiographic lesions in a pulmonary tuberculosis (PTB) patient cohort before and 60 days after anti-tuberculous treatment (ATT) initiation.

**Methods:**

Circulating levels of cytokines (IL-2, IL-4, IL-6, IL-10, IFN-γ, TNF-α) and C-reactive protein (CRP), as well as values of erythrocyte sedimentation rate (ESR) were measured in cryopreserved serum samples obtained from 73 PTB patients at pre-ATT and day 60 of treatment. Changes of the immune profile over time were compared with mycobacterial loads in sputum and culture conversion at day 60 of ATT. Additional analyses tested associations between improvement of chest radiographic lesions at day 60 and pre-treatment status of inflammation and mycobacterial loads.

**Results:**

Within the inflammatory parameters evaluated, values of CRP, IL-2, IL-4, TNF-α and ESR significantly decreased upon treatment initiation. On the converse, IL-10 levels substantially increased at day 60 of ATT, whereas concentrations of IL-6 and IFN-γ remained unchanged. Multidimensional analyses revealed that ESR, IL-2, IL-4 and CRP were the parameters with the highest power to discriminate individuals before and after treatment initiation. We further demonstrated that higher bacterial loads in sputum at pre-ATT were associated with increased systemic inflammation and higher risk for positive *M. tuberculosis* sputum cultures at day 60 of treatment. Furthermore, we found that pre-ATT mycobacterial loads in sputum and systemic inflammation synergistically associated with the status of radiographic lesions during treatment (Relative risk for chest X-ray improvement: 10.0, 95 % confidence interval: 2.4–40.0, *P* = 0.002).

**Conclusions:**

*M. tuberculosis* loads in sputum are directly associated to the status of systemic inflammation and potentially impact the immune profile, culture conversion and evolution of lung lesions upon ATT initiation.

**Electronic supplementary material:**

The online version of this article (doi:10.1186/s12879-016-1736-3) contains supplementary material, which is available to authorized users.

## Background

Upon *M. tuberculosis* infection, a host can develop a wide range of disease manifestations ranging from asymptomatic infection to severe progressive disease [1]. Importantly, only 10 % of individuals exposed to *M. tuberculosis* develop active disease, which highlights the importance of understanding the key determinants of susceptibility to infection. The determinants of the TB clinical presentation are described to involve a complex relationship between the mycobacterium and the host immune responses [2, 3]. Successful host response against *M. tuberculosis* requires the production of pro-inflammatory cytokines including IFN-γ and TNF-α [4, 5]. Indeed, individuals genetically deficient in molecules from the IFN pathway, as well as those under treatment of chronic conditions with TNF-α blockers, are highly susceptible to severe TB [6].

Existing blood-based tests, such as IFN gamma release assays (IGRA) are inadequate for monitoring treatment response [7]. Therefore, host biomarkers for monitoring treatment response have been considered as important priorities for TB research [8].

It has been recently described in distinct TB patient cohorts from Brazil and India that TB disease severity is associated elevated circulating levels of CRP and other pro-inflammatory cytokines and lipid mediators [9, 10]. Although there have been indications from experimental models that *M. tuberculosis* loads in the lungs are associated with the inflammatory profile and lung disease severity [11, 12] this relationship has not been yet fully investigated prospectively.

The present study addresses this question in a cohort of PTB patients from a highly endemic area in Brazil. The findings argue that there is a strong relationship between pre-treatment mycobacterial loads and systemic inflammation, which may set the stage for persistence of sputum culture positivity and radiographic improvement after 2 months of antibiotic chemotherapy.

## Methods

### Study design

We performed a longitudinal cohort study involving 73 patients diagnosed with PTB and admitted to a referral hospital for the treatment of TB in the state of Rio de Janeiro, Brazil (Instituto Estadual Ary Pareiras), between March 2007 and August 2009. We included subjects from both sexes, infected or not by HIV, with positive smear microscopy and culture for *M. tuberculosis* complex subsequently confirmed by biochemical tests. We excluded subjects aged under 18 and over 60 years; taking anti-TB drugs before admission, with insulin-dependent diabetes mellitus, renal failure or hemodialysis and peritoneal dialysis blood transfusion; women in pregnancy or lactation period; and those whose clinical samples were not subjected to bacteriological or laboratory tests. The outcome in our study was to evaluate the frequency of negative culture at day 60 of treatment and its association with bacteriological status, inflammatory profile and radiological aspects observed at pre-ATT and day 60 of ATT. Our study population was followed up very closely to assure treatment adherence. During the study period, all pulmonary TB patients included remained hospitalized and received anti-TB drugs under directly observed manner. For quality control purposes, we measured each inflammatory parameter in 10 age- and sex-matched healthy individuals. This control group was composed by health care professionals from our laboratory who were screened negative from TB by means of negative Tuberculin Skin Test (TST), negative IGRA assay, no symptoms and no lesions in chest X-ray.

### Radiographic evaluation

All patients underwent chest radiography in the pre-ATT and day 60 in posterior-anterior (PA) and left profile (LP). To improve the assessment of the quality of images evaluated in this study, radiographs were digitized at the Advanced Nuclei for High Performance Computing, Federal University of Rio de Janeiro, Brazil. Once scanned and saved, the images were viewed analyzed by a two pulmonologists (E.M., A.K.), who were blinded to the subjects clinical/demographic characteristics, laboratory results and treatment outcome. A standardized case report form was used to document the presence of following images, according to the chest radiographs of the Committee of the Fleischner Society [13]: volume loss, cavitation, consolidation, nodules, infiltrates, hilar lymph node enlargement, pleural effusion, pleural thickening, lung sequel and bronchiectasis. The location of consolidation or cavities was specified dividing the lung into six zones (upper/middle/lower for each lung). As a quality check, the senior radiologist (E.T.) read each film, also blinded to original interpretations, when discordance occurred between two pulmonologists. Thus, every third was analyzed for the presence of the changes at pre-ATT and day 60. The ratings were categorized as: (i) improvement or evolving to the sequel and; (ii) no improvement when remained the disease activity or lesions were suggestive of fibrosis and scarring.

### Immunoassays

The serum levels of IL-2, IL-4, IL-6, IL-10, TNF-α and IFN-γ were measured using a pre-determined multiparametric cytometry-based bead assay (Cytometric Bead Array Human Th1/Th2 Cytokine Kit, BD Biosciences, San Jose, CA) following the manufacturer’s protocol. This assay provides a method of capturing a set of analytes with beads of known size and fluorescence, making it possible to detect analytes using flow cytometry (FACSCalibur; BD Biosciences) [14–16]. The concentrations of samples were calculated by extrapolating the mean fluorescence intensity (MFI) on the respective standard curves. A single flow cytometry operator performed the assays. Serum levels of CRP were determined using an ELISA-based assay (Ebioscience, San Diego, CA). Values of ESR were quantified at the clinical laboratory of the Federal University of Rio de Janeiro using the Westergren method. The limits of detection of the cytokines (in pg/mL) were: IL-2: 11.2, IL-4: 1.4, IL-6: 1.6, IL-10: 0.13, TNF-α: 1.2, IFN-γ: 0.8. The limit of detection for CRP was 0.15 mg/L.

### Data analysis

Median and interquartile ranges (IQR) were used as measures of central tendency. Levels of inflammatory markers were compared in the study population (and subgroups) between pre-ATT and day 60 of treatment using the Wilcoxon matched pairs test with Holm-Bonferrori adjustment for multiple comparisons. Correlations between the markers were assessed using the Spearman rank test. Inferential network analysis was performed using the statistically significant correlations observed in the Spearman correlation matrices as previously described [17–20]. Briefly, each biomarker is selected as a target, and the software (JMP 12.0, SAS, Cary, NC, USA) performs a search within the other mediators for those that are correlated, with the target calculating a correlation matrix using Spearman rank tests. As a result, the features related to the selected target are linked. The links shown in the networks represent statistically significant Spearman rank correlations (*P* < 0.05) after adjustments for multiple measurements (Holm-Bonferrori’s method). Moreover, two models of principal component analysis (PCA), as described previously [9, 21] were employed to test which combination of inflammatory markers could better distinguish individuals before and after ATT initiation. Hierarchical cluster analyses with bootstrap (100×) were performed to describe the overall expression profile of inflammatory markers in different settings. Comparisons of proportions between study groups were tested using the Fisher’s exact test. The same test was used to compare frequency of patients displaying improvement in chest X-ray lesion evaluation. Logistic regression analyses adjusted for age and gender were employed to verify the associations between indicated variables and culture positivity or radiographic improvement at day 60. All analyses were pre-specified. Two-sided *p* values of <0.05 after adjustment for multiple comparisons were considered statistically significant. Statistical analyses were performed using SPSS 20.0 (IBM statistics), Graphpad Prism 6.0 (GraphPad Software, San Diego, CA) and JMP 12.0.

## Results

### Defining a biosignature of serum inflammatory markers in TB patients undergoing ATT

A total of 73 PTB patients were prospectively studied (median age: 41 years, interquartile range [IQR]: 29–50; male 84 %; Table 1). The median hemoglobin value was 11.6 g/dL (IQR: 9.9–12.9 g/dL). Cavitary lesions were identified in 42 patients (57.5 %). Multiple cavitations (>1) were observed in 25 individuals (34.2 %). The vast majority of the study population referred smoking history (74.1 %) and alcoholism (72.6 %). Approximately 1/3 reported illicit drugs use (31 %). HIV seropositivity was 13 % (Table 1).

**Table 1:** Characteristics of the study participants

Characteristic	
*N*	73
Male – no. (%)	49 (84.48)
Median age – y (IQR)	41 (29–50.3)
Median hemoglobin – g/dL (IQR)	11.6 (9.9–12.9)
AFB+ sputum smear	52 (71.2)
Cavitary lung lesion – no. (%)	42 (57.5)
Smoking history – no. (%)	43 (74.13)
Alcoholism – no. (%)	53 (72.6)
Illicit drugs use – no. (%)	18 (31.03)
HIV/AIDS – no. (%)	8 (13.79)
Fever – no. (%)	39 (67.24)
Cough – no. (%)	45 (77.58)
Anemia – no. (%)	63 (86.3)
Hemoptysis – no. (%)	13 (22.41)

*AFB* acid-fast bacilli, *IQR* interquartile range

Anemia criteria was based on hemoglobin value below 13.5 g/dL for men or 12.0 g/dL for women

**Fig. 1:**
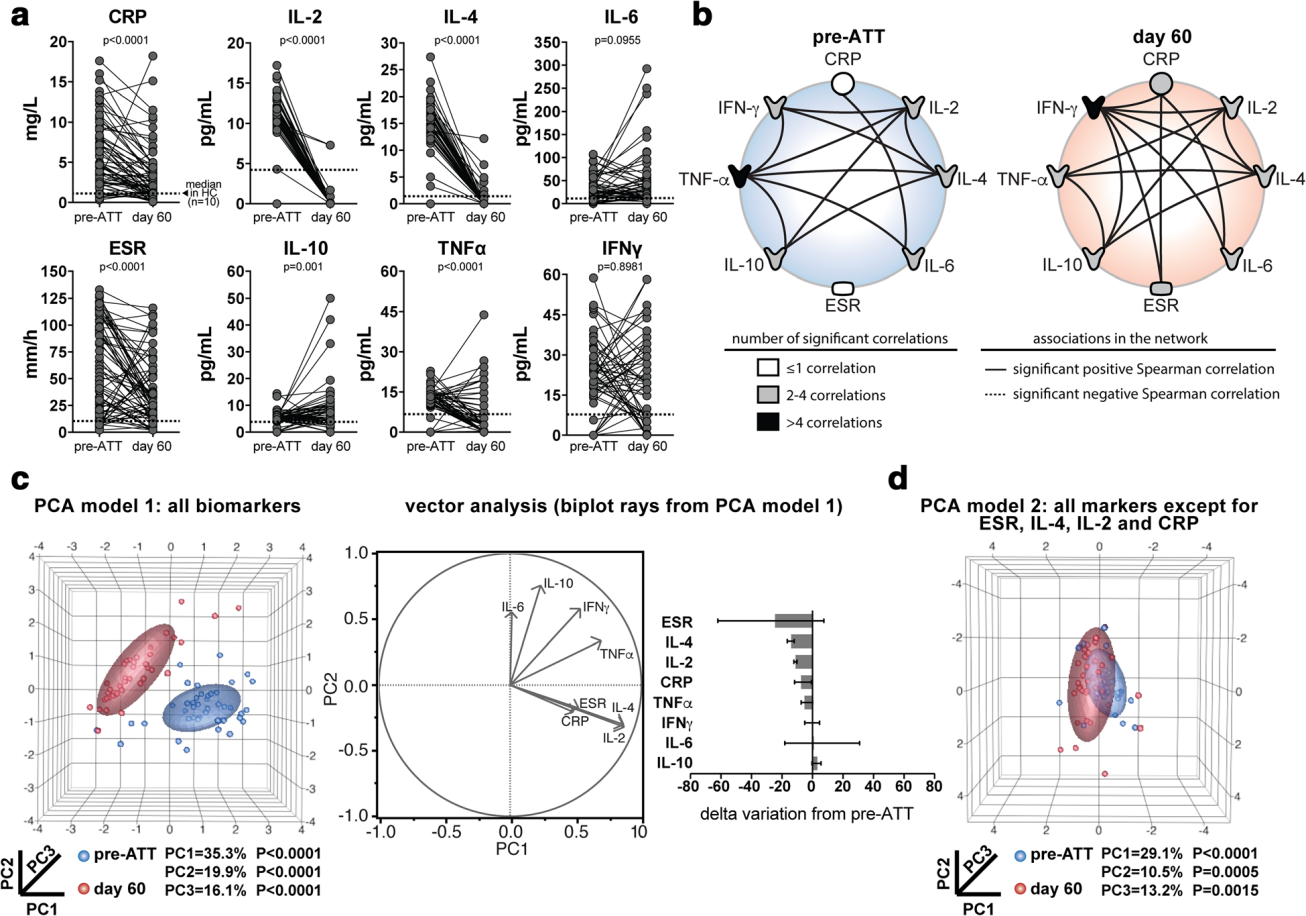
Prospective immune profiling of pulmonary TB patients before and 60 days after anti-mycobacterial treatment initiation. **a** Levels of C-reactive protein (CRP), indicated cytokines and erythrocyte sedimentation rate (ESR) were measured in serum samples from 73 patients with pulmonary TB at day 0 and 60 of anti-tuberculous treatment (ATT). Data were analyzed using Wilcoxon matched pairs test. Dotted lines represent median values detected in age- and gender-matched uninfected and healthy controls (*n* = 10), to serve as reference. **b** Network analysis was performed based on Spearman correlation matrices as described in Methods. Each connecting line represents a statistically significant correlation (*p* < 0.05). The strength (*r* value) and level of significance (*p*-value) for each significant correlation is shown in Additional file 1: Table S1. **c** A model of principal component analysis (PCA) was employed to test whether combination of the markers could cluster the patients at different time points of ATT (*left panel*). Vector analysis was further utilized to illustrate the influence of each marker in data distribution of the PCA model (*center panel*). Delta variation of the indicated markers from day 0 to day 60 of ATT initiation (*right panel*). Data represent median and interquartile ranges. **d** A second model of PCA in which all variable were included except from those with the largest delta variation from day 0 to day 60 of ATT was designed. HC = healthy controls, PC = principal component

An exploratory analysis examined circulating levels of inflammatory parameters, including cytokines such as IL-2, IL-4, IL-6, IL-10, TNF-α, IFN-γ, as well as CRP and ESR. We observed that CRP, IL-2, IL-4, TNF-α and ESR values significantly decreased whereas concentrations of IL-10 increased upon ATT initiation (Fig. 1a). Serum levels of IL-6 and IFN-γ did not substantially change between pre-ATT and day 60 of treatment (Fig. 1a). We have recently shown that the statistical relationships between inflammatory markers using network analysis can help define the profile of the inflammatory milieu in different disease models [17–20]. In the present study, we detected that the overall profile of the correlations between the serum markers changed between days 0 and 60 of anti-microbial therapy (Fig. 1b). Notably, there were no significant negative correlations between the parameters examined in this analysis. Moreover, at pre-ATT, TNF-α was the marker that exhibited the highest number of significant correlations with the other parameters (Fig. 1b and Additional file 1). Intriguingly, amongst all the cytokines evaluated at this time point, only IL-6 was significantly correlated with CRP (*r* = 0.44, *p* = 0.003), and no robust association was observed between the biomarkers and ESR (Fig. 1b). These finding suggest that the cytokine inflammatory milieu was not directly correlated with values of acute phase parameters in PTB patients before treatment initiation. Upon ATT, the most important node in the network of associations was IFN-γ (Fig. 1b). Interestingly, at day 60 of treatment, IFN-γ levels became positively correlated with CRP (*r* = 0.39, *p* = 0.009) and ESR (*r* = 0.38, *p* = 0.01), arguing that individuals on ATT who remained with higher levels of this cytokine exhibited increased systemic inflammation.

We next employed a PCA model inputting values of the cytokines and acute phase parameters and observed that the overall pattern of systemic inflammation could distinguish PTB patients at the different study time points (Fig. 1c). A complementary vector analysis revealed that ESR, IL-4, IL-2 and CRP were the most important factors determining the discrimination power of the PCA model (Fig. 1c). To confirm this result, we designed a second PCA model including all the parameters except for ESR, IL-4, IL-2 and CRP. In this new model, patients grouped according to the study time point were no longer distinguishable (Fig. 1d).

### Higher mycobacterial loads in sputum smears are associated increased risk for culture positivity at day 60 of ATT

**Fig. 2:**
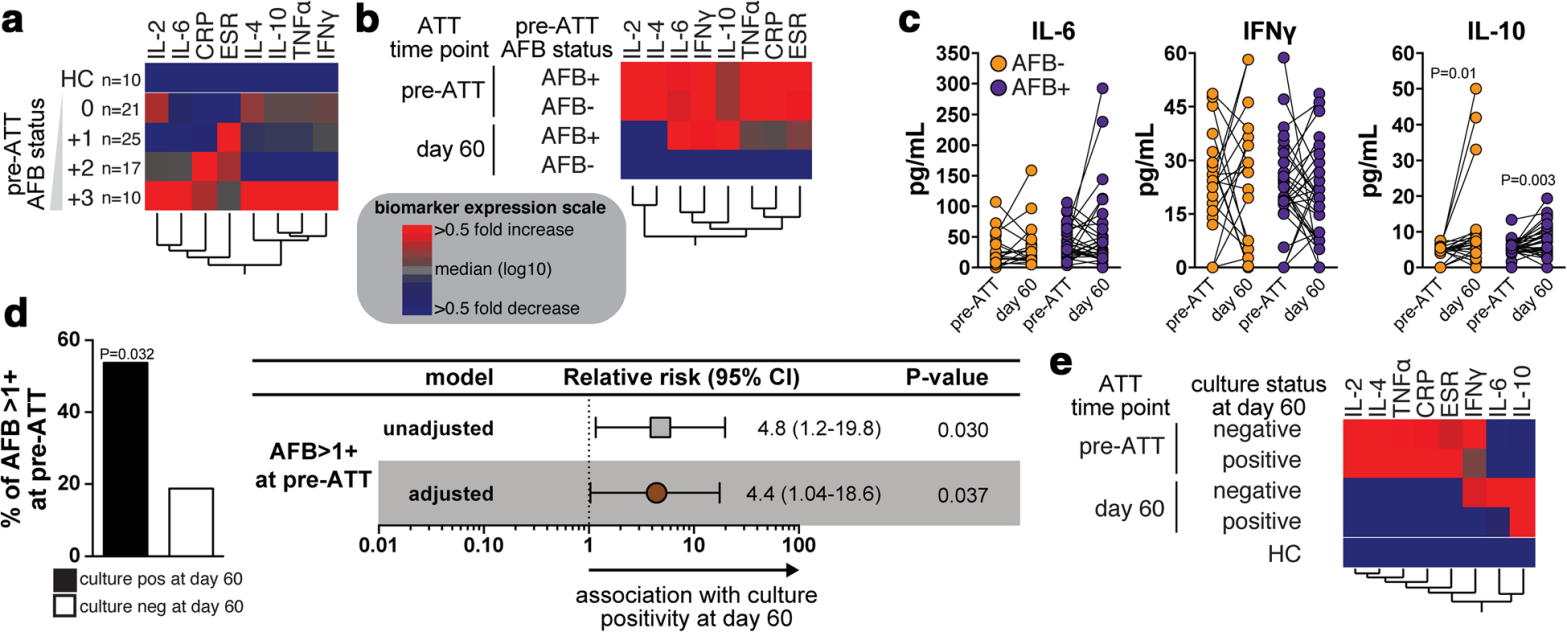
Inflammatory profile, mycobacterial loads and culture positivity at day 60 of antimicrobial treatment in PTB patients. **a** A hierarchical clustering analysis (Ward’s method with bootstrap) was employed to show the immunological profile of TB patients with different AFB status in smear samples at pre-ATT. Data was processed as described in Methods. In the heatmap, the squares represent values below or above the median values (log10) of a given biomarker in the entire study population (*n* = 73). **b** The same hierarchical clustering analysis was performed to compare the inflammatory profile of TB patients stratified according to the AFB status in sputum smears at pre-ATT and the study time points. **c** Serum levels of the indicated cytokines were compared between day 0 and day 60 of ATT in TB patients presenting with positive or negative AFB in sputum smears at study enrollment. Data were analyzed using the Wilcoxon matched pairs test. Significant *p*-values are shown. **d** Left panel shows frequency of TB patients with AFB >1+ in sputum smears within the groups of individuals stratified by culture status at day 60 post ATT initiation (positive culture at day 60, *n* = 13; negative culture, *n* = 53). Data were compared using the Fisher’s exact test. Right panel shows logistic regression analysis adjusted for age, gender and hemoglobin levels was performed to determine the association between AFB sputum smear > +1 at pre-ATT and culture positivity at 60 day upon ATT initiation. **e** A hierarchical clustering analysis was employed to illustrate the inflammatory profile of TB patients stratified by treatment time point and culture status at day 60 after ATT initiation

Previous studies have suggested that presence of *M. tuberculosis* in sputum is associated with more severe clinical forms of TB, which is reflected by elevated levels of acute phase reactants [10]. To test if the mycobacterial loads in sputum are associated with systemic inflammation in our patient cohort, we performed a hierarchical cluster analysis of the serum levels of the inflammatory markers as well as values of ESR in patients stratified by acid-fast bacilli (AFB) smear grade at pre-ATT. We confirmed that patients with higher mycobacterial loads in sputum smears indeed exhibit a distinct pro-inflammatory signature profile (Fig. 2a). Interestingly, CRP values were the highest in individuals with AFB 2+ whereas ESR values were higher in those with AFB 1+, leading us to speculate that the relationships observed between mycobacterial loads in sputum and circulating cytokine levels may not be strictly representing the degree of systemic inflammation and may rather highlight qualitative differences in inflammation and/or immune response. Additional analyses using Spearman correlation ranks tested whether each individual inflammatory parameter are correlated with the AFB smear grade values. Of note, amongst all the parameters, only CRP (*r* = 0.28, *p* = 0.04), IL-6 (*r* = 0.33, *p* = 0.035) and IL-10 (*r* = 0.39, *p* = 0.02) were modestly associated with mycobacterial loads in sputum. Furthermore, we prospectively assessed changes in the inflammatory biosignature profile from day 0 to day 60 of ATT in patients presenting with positive or negative AFB staining at the study enrollment. Hemoglobin concentrations consistently increased after implementation of anti-tuberculous therapy (median 12.8 g/dL, IQR: 11.4–14.2 on day 60 of ATT vs. median 11.6 g/dL, IQR: 9.9–12.9 on pre-ATT, *p* = 0.0004), indicating that antibiotic treatment successfully reduced the degree of anemia in the patient population. As expected, the values of the inflammatory markers were markedly decreased at 60 days of antimicrobial treatment (Fig. 2b). In addition, at the study enrollment time point, the smear status did not associate with significant changes expression profile of the biomarkers (Fig. 2b). Upon treatment initiation, we observed that individuals who were AFB+ at pre-ATT remained with heightened levels of IL-6, IL-10 and IFN-γ compared to those with negative smears (Fig. 2b, c). Of note, within each group of patients (AFB− or AFB+), the circulating levels of both IL-6 and IFN-γ did not significantly change upon treatment initiation, whereas IL-10 levels were substantially increased in both groups (Fig. 2c).

We next tested whether mycobacterial loads in sputum smears could also reflect early treatment responses in our patient population. We found that individuals who remained with positive *M. tuberculosis* sputum cultures at day 60 of treatment exhibited more frequently higher AFB smear grades at pre-ATT than those who experienced culture conversion (53.8 % of those with culture positive at day 60 had AFB > 1+ at pre-ATT vs. 18.8 % of those with culture negative at day 60, *p* = 0.032; Fig. 2d). Logistic regression analysis adjusted for age, gender and hemoglobin levels confirmed that an AFB smear grade above 1+ was indeed associated with increased risk for *M. tuberculosis* culture positivity at day 60 of ATT (Relative risk [RR] = 4.4; *p* = 0.037; Fig. 2d). To prospectively depict the serum inflammatory profiles of PTB patients diverging in early ATT responses, we performed an additional hierarchical cluster analysis of the biomarker values at day 0 and 60 of treatment initiation. This approach revealed that *M. tuberculosis* culture positivity after treatment initiation was associated with sustained heightened circulating levels of IL-10 only, whereas IL-6, IL-10 and IFN-γ remained at higher values in the group of patients who had negative cultures at day 60 of ATT (Fig. 2e). Of note, within this study population, 3 patients ended up exhibiting drug resistance (all of those were rifampicin resistant). Only 1 out of these 3 patients remained sputum positive at day 60 of treatment. Our conclusion in the context of this study is thus that the lack of early sputum clearance detected in some patients evaluated here was not directly related to drug resistance. Nevertheless, this specific conclusion is weakened by the very low numbers of drug resistant cases evaluated in the present study.

### Cavitary lung lesions and cytokine levels, AFB smear status, sputum cultures and radiographic improvement of lung disease in patients undergoing ATT

**Fig. 3:**
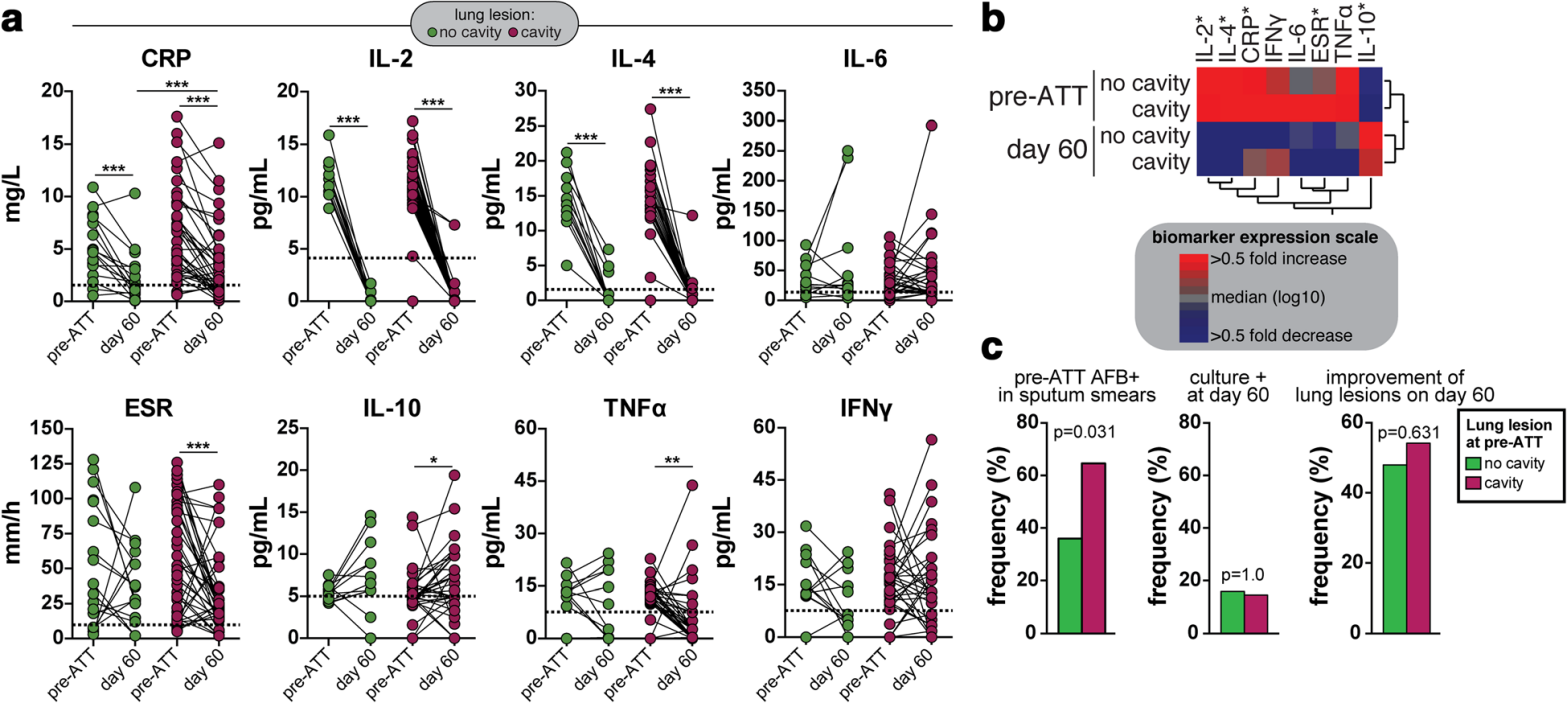
Association between cavitary lung disease, systemic inflammation and radiological improvement of TB lung lesions. **a** Levels of C-reactive protein (CRP), indicated cytokines and erythrocyte sedimentation rate (ESR) were compared between day 0 and 60 of anti-tuberculous treatment (ATT) in TB patients with (*n* = 42) or without (*n* = 31) cavitary disease. The Wilcoxon matched pairs test was used to compare changes in the biomarker levels between the timepoints in each clinical subgroup. The Mann–Whitney test was used to compare biomarker values between cavitary vs. non-cavitary TB disease at different timepoint. Dotted lines represent median values detected in age- and gender-matched uninfected and healthy controls (*n* = 10), to serve as reference. * *p* < 0.05; ** *p* < 0.01; *** *p* < 0.001. **b** A hierarchical clustering analysis (Ward’s method with bootstrap) was employed to illustrate the inflammatory profile of TB patients stratified by treatment timepoint and cavitary lung disease at pre-ATT. Asterisks highlight the parameters that exhibited statistically significant differences in one or more comparison [shown in (**a**)]. **c** frequency of TB patients with or without cavitary TB exhibiting AFB+ smears at pre-ATT (*left panel*), positive *M. tuberculosis* sputum culture (*center panel*) and improvement of lung lesions assessed by chest X-rays at day 60 of treatment.. Data were compared using the Fisher’s exact test

Cavitary lung disease increases TB and is described to contribute to antibiotic failure and the emergence of antibiotic resistance [22–24]. Given the fact that up to 57.5 % of our study population exhibited lung cavitations on radiographic evaluation at the enrollment (Table 1), we next examined the changes in the biomarker serum concentrations upon ATT initiation in TB patients stratified by cavitary disease. Of note, patients with cavitary disease were similar to those with non-cavitary TB with regard to age, gender, prevalence of symptoms, tobacco use, alcoholism history, HIV status and hemoglobin levels levels (all with *p* > 0.05). Levels of CRP, IL-2 and IL-4 consistently decreased n both groups of patients with cavitary and non-cavitary disease (Fig. 3a). In addition, at day 60 of ATT, CRP concentrations were found to be higher in patients with pre-ATT cavitary lesions compared to those with non-cavitary disease (Fig. 3a). Furthermore, both IL-6 and IFN-γ levels remained unchanged upon treatment initiation regardless of the cavitary disease status (Fig. 3a). Values of ESR as well as concentrations of TNF-α substantially decreased, whereas levels of IL-10 increased, only in the subset of patients exhibiting cavitary disease at pre-ATT (Fig. 3a). Aside from the CRP levels, we observed no statistically significant differences in the remaining biomarker values between patients with cavitary lesions and those with non-cavitary disease at day 60 of ATT. Hierarchical cluster analyses of the median values of each biomarker per study group revealed that TB patients summarized these findings (Fig. 3b). Notably, whilst we detected increased frequency of pre-ATT AFB+ in sputum smears from individuals with cavitary disease vs. those with no lung cavitation (Fig. 3c), these clinical groups were undistinguishable with regard to frequency of *M. tuberculosis* culture positive and radiographic improvement of lung lesions at day 60 of ATT (Fig. 3c).

### Radiographic improvement of lung disease in TB patients upon ATT initiation is associated with pre-treatment levels of C-reactive protein and AFB smears in sputum

**Fig. 4:**
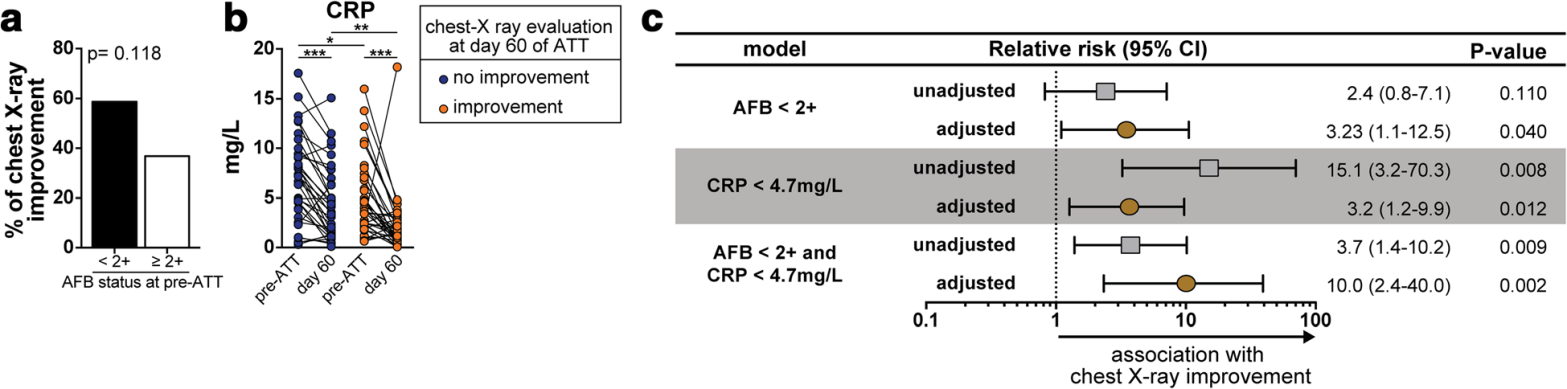
Association between mycobacterial loads in sputum, systemic inflammation and radiological improvement of TB lung lesions. **a** Frequency of individuals exhibiting radiological improvement of lung lesions assessed by chest x-ray in the groups of TB patients according to the indicated AFB status in smear samples at pre-ATT. Data was analyzed using Fisher’s exact test. **b** Serum levels of CRP were compared between day 0 and day 60 of ATT in TB patients stratified by improvement lung lesions assessed by chest X-ray. Data were analyzed using the Wilcoxon matched pairs test. * *p* < 0.05; ** *p* < 0.01; *** *p* < 0.001. **c** Different models of regression analysis adjusted for age, gender and hemoglobin levels were employed to determine the association between low AFB sputum smear grade (<2+), low serum levels of CRP (concentrations below the median value of the entire study population, 4.7 mg/L) at pre-ATT and radiological improvement at day 60 of ATT

Radiographic assessment of PTB patients during antimicrobial treatment has been used as a tool to estimate disease activity [25]. In the present study, the vast majority of the patient population, (52 %) exhibited significant radiographic improvement (38 out of 73 individuals) assessed by a panel independent physician experts. Individuals displaying lower AFB smear grades (AFB < 2+) at pre-ATT tended to present with radiographic improvement of lung disease compared to those with higher mycobacterial loads in sputum, however this difference was not statistically significant (*p* = 0.118; Fig. 4a). Among all the inflammatory markers examined, only CRP values exhibited statistically significant prospective changes in patients stratified according to radiographic improvement at day 60 of ATT. We observed that CRP levels decreased in PTB patients undergoing ATT independently of the status of the radiographic evaluation at day 60 (Fig. 4b). Moreover, at day 60 of treatment, patients who had chest X-ray improvement exhibited lower CRP serum concentrations than those who did not (Fig. 4b). We next performed a logistic regression adjusted for age, gender and hemoglobin levels to examine the association between lower mycobacterial loads in sputum, lower concentrations of CRP or both at pre-ATT and the odds for radiographic improvement at day 60 of treatment. Importantly, we detected a synergistic effect of low AFB counts and low CRP values on odds for improvement in lung disease (Fig. 4c). Individuals presenting with pre-ATT AFB smear < 2+ and CRP levels < 4.7 mg/L were ten times more likely to exhibit radiographic improvement of lung disease compared to those with higher values of these parameters (Relative risk: 10.0, 95 % CI: 2.4–40.0; *p* = 0.002; Fig. 4c).

## Discussion

Several exploratory studies have evaluated the diagnostic potential of cytokine biomarkers other than IFN-γ for monitoring anti-tuberculous therapy [2, 3], but no prospective study have measured simultaneously the direct associations between mycobacterial loads, Th1/Th2 cytokines, acute phase parameters and pulmonary lesions upon treatment initiation, as we performed in our longitudinal study. A recent systematic review by Clifford et al. [26] identified only 12 longitudinal studies that measured ex vivo serum cytokine concentrations without a stimulation step during anti-TB treatment. The largest studies were those by Azzuri et al. [27] and Wang et al. [28], with 220 and 87 TB cases, respectively. Thus, additional prospective studies assessing effects of ATT on the host inflammatory profile are greatly needed, especially in endemic areas such as Brazil.

In the present study, we prospectively depict the immune profile, microbial clearance and evolution of radiographic lesions in 73 pulmonary TB patients before and 60 days after anti-tuberculous treatment initiation. Logistic regression analysis adjusted for age, gender and hemoglobin levels confirmed that an AFB smear grade above 1+ was indeed associated with increased risk for *M. tuberculosis* culture positivity at day 60 of ATT. These results confirm that monitoring sputum and culture conversion may be a good indicators of treatment outcome for drug-sensitive TB but may present low sensitivity and modest specificity for predicting failure and relapse [29]. In our study, we found that pre-ATT mycobacterial loads in sputum and systemic inflammation synergistically associated with the status of radiographic lesions during treatment. This approach could potentially improve the predictive value of non-favorable outcomes associated with ATT in future clinical trials.

Within the inflammatory parameters evaluated, values of CRP, IL-2, IL-4, TNF-α and ESR significantly decreased upon treatment initiation similarly to other studies [28, 30–36]. On the converse, IL-10 levels substantially increased at day 60 of ATT, as also described elsewhere [5, 27, 28, 30, 33–40]. Nevertheless, the reports on the changes in IL-10 levels are conflicting, as other studies have described decreases of the systemic levels of this cytokine during ATT [5, 27, 28, 35]. It is possible that differences in study populations as well as distinct epidemiological/genetic background could explain these conflicting findings.

In our study, concentrations of IL-6 and IFN-γ remained unchanged during the anti-TB treatment in disagreement with different investigations where IL-6 and IFN-γ tended to decrease during ATT [28, 30, 31, 33, 36–38] and with other reports which showed increases in IFN-γ levels [39, 41–43]. Additional studies systematically investigating patients from different ethnicity/genetic backgrounds should be performed to narrow down the mechanisms underlying these discrepancies. Importantly, at day 60 of treatment, the cytokine network of associations based on Spearman ranks revealed that the cytokine exhibiting the higher number of significant correlations with the remaining biomarkers was shifted from TNF-α to IFN-γ. It is possible that these two cytokines may be critical for driving activation of specific immune pathways evoked by ATT; however, this hypothesis requires additional mechanistic studies to be tested.

Multidimensional analyses revealed that ESR, IL-2, IL-4 and CRP were the parameters with the highest power to discriminate individuals before and after treatment initiation. As highlighted previously [44], our findings indicate that monitoring acute phase parameters (ESR, CRP), Th1 cytokines (IL-2) and anti-inflammatory (IL-4) may help understanding the multidimensionality of immunopathology in TB and point to novel potential therapeutic targets for pulmonary TB. Recently, Mihret et al. [39], while evaluating HIV negative and positive TB cases, have demonstrated that the ratios of IFN-γ/IL-10 and IFN-γ/IL-4 display significant increases after treatment in HIV negative TB cases but not in HIV positive TB cases.

Remarkably, we observed that individuals who were AFB+ at pre-ATT remained with heightened levels of IL-6, IL-10 and IFN-γ upon treatment initiation compared to those with negative smears, similarly to that described by Djoba et al. [33] and Chowdhury et al. [37] where serum levels of IFN-γ, TNF-α, IL-6 and IL-10 were shown to be directly associated with the bacterial load. As expected, the values of the pro-inflammatory markers were markedly decreased at 60 days of antimicrobial treatment. Our results also revealed that there is a strong association between pre-treatment mycobacterial loads and risk for culture positivity at day 60 of ATT initiation similarly to results described by Djoba et al. [33].

The apparent association between increased levels of only IL-10, among all the other markers, and presence of *M. tuberculosis* culture positivity at day 60 of ATT is intriguing and may underline a potential inability of the immune system to clear the bacteria from the lung. Indeed, IL-10 induction is described to be a potential scape mechanism used by *M. tuberculosis* to survive in macrophages [45]. *M. tuberculosis* can be recognized by TLR-2 and induce production of IL-10, that promotes differentiation into a M2-like macrophage phenotype and possibly Th2 adaptive responses [46]. Moreover, recent studies demonstrated a direct association between TLR2 polymorphisms and enhanced bacterial loads in pulmonary tuberculosis patients [45]. Our group is currently assessing the influence of immune related polymorphisms in cytokine levels in TB patients undergoing ATT.

More recently, some studies have reported the association between sputum conversion at 60 day of TB treatment with decreases in serum levels of CRP, CXCL10, IL-17, MMP-8, as well as with cytokine responses after *M. tuberculosis*-specific in vitro stimulation of peripheral blood. Moreover, sputum conversion has also been associated with decreases in IFN-γ, TNF-α and IL-2 levels as well as in frequency of IFN-γ^+^CD4^+^ T-cells that express immune activation markers CD38 and HLA-DR and the proliferation marker Ki-67 in peripheral blood [32, 34, 46–49]. Whether our findings reflect changes in T-cell compartment or in other immune cells deserves further investigation.

In the present study, we detected an increased frequency of AFB+ smears at pre-ATT in patients presenting with cavitary lesions. This finding is consistent with the idea that cavitary lesions increase risk for TB transmission [22, 24]. Interestingly, bilateral cavity formation is thought to be the most significant predictor of treatment failure for extensively drug-resistant TB [23]. Our current understanding of the mechanisms implicated in cavity formation is still elusive and derive mostly from experimental models and observational studies of human specimens [50, 51]. We observed that the circulating levels of all the parameters assessed could not distinguish patients with non-cavitary disease from those presenting with cavitary lung lesions at pre-treatment. In addition, the magnitude of changes in levels of IL-2, IL-4, IL-6 and IFN-γ between day 0 and 60 of ATT was similar between the groups of patients stratified according to cavitary disease status. Although CRP concentrations consistently decreased at day 60 of ATT in both subgroups of patients, individuals with cavitary disease exhibited heightened values compared to patients with non-cavitary TB. Furthermore, serum levels of IL-10 significantly increased while TNF-and ESR values substantially decreased from day 0 to day 60 of ATT in patients with cavitary TB but not in those with non-cavitary lung disease. These differential changes detected may indicate that individuals with lung cavities exhibit a slightly different inflammatory response to ATT. However, these disparities in biomarker levels may not be biologically relevant as they did not reflect differences in early treatment responses, as the frequencies of patients presenting with culture positive or improvement of lung lesions by radiographic evaluation on day 60 of ATT were similar between the subgroups with or without cavitary TB. It is possible that de directly observed therapy employed here, together with the close monitoring of patients who remained in the hospital for the first 60 days of treatment, resulted in good treatment response regardless of cavitary disease.

Individuals presenting with pre-ATT AFB smear < 2+ and CRP levels < 4.7 mg/L were more than 12 times more likely to exhibit radiographic improvement of lung disease. Few studies have evaluated the interaction within bacterial load, inflammation response and radiographic changes [33]. Nevertheless, other studies have reported associations between a number of cytokines, such as TNF-α, IL-1β, IL-6, IL17 and TGF-β1, and radiographic TB lung disease presentation [30, 33, 36, 48, 52]. CRP is acute inflammatory protein whose production by hepatic cells is stimulated by TNF-α, IL-1 and IL-6. This protein has important functions, which include induction of inflammatory responses and participation in immune activation following infection. Many authors suggest that a fine control of inflammatory responses is crucial to promote clinical and microbiological improvement of TB patients [8, 10, 53]. Exhaustive inflammatory responses lead to immunopathology-driven pulmonary injury, which directly impacts disease severity in pulmonary tuberculosis [12, 54]. Lower CRP concentrations found in a subset of TB patients prior to ATT may be indicative of a mild/moderate systemic inflammatory response reflected by increase odds for improvement in chest-X-ray 60 days after treatment initiation.

The strengths of our study include: a) cohort of hospital-based pulmonary TB patients that assessed simultaneously the direct associations between mycobacterial loads, Th1/Th2 cytokines, acute phase parameters and pulmonary lesions upon treatment initiation, b) our study population was followed up very closely to assure treatment adherence: during the study period, all pulmonary TB patients remained hospitalized and received anti-TB drugs under directly observed manner, c) the personnel performing the laboratory and chest radiographs analysis were unaware of the treatment outcomes, d) innovative statistical analyses employing multidimensional approaches were used to define a biosignature that better relates to the clinical outcomes assessed.

Our study has some limitations. We did not evaluate other diseases, which could result in similar disease presentation than TB. Identification of a biosignature distinguishing TB from other clinical disease groups has been published by us and others [9, 10, 55–57] and it was not an aim of the present study. Our study has a very narrowed goal of testing associations between bacillary loads, systemic inflammation and radiological changes in individuals with active TB undergoing treatment. Moreover, we have not screened our patients for a number of co-infections, such as viral hepatitis and helminth infections, as well as for comorbidities such as steatohepatitis, which could affect immune responses in TB patients [58, 59]. Additional studies with larger numbers of patients will be required to investigate whether the results described here in terms of prospective changes in inflammatory biomarkers in the context of ATT are considerably affected by these co-morbidities. We have assessed serum levels of inflammatory markers and have not studied specific cellular immune responses against *M. tuberculosis* infection. The use of serum samples may result in lower specificity than measuring cytokine responses induced by stimulations with TB antigen in cell cultures. Few HIV positive TB cases were included in the analysis, due to the lower prevalence of TB-HIV co-infection in our study setting. Finally, we did not evaluate different patterns of the host immune response according to different *M. tuberculosis* lineages [60]. Nevertheless, our results were able to demonstrate unique associations between host inflammatory markers in serum and microbiological and radiological outcomes after the onset of anti-TB treatment. Due to the relatively small number of patients studied in our exploratory study, it would be important to validate the results reported here in additional cohorts with larger patient populations as well as in different epidemiological settings.

## Conclusions

In the present study, we identified host biomarkers that could potentially be used in addition to the clinical evaluation of TB patients to estimate sputum clearance and evolution of radiographic lung lesions upon initiation of anti-TB treatment. The relationship between CRP with bacterial loads in sputum and chest-X-ray deserves future mechanistic investigation. Additionally, our findings indicating a direct relationship between higher bacterial loads sputum at pre-ATT and no improvement of chest-X-ray at day 60 of treatment argue that there was a lack of mycobacterial control in a subset of patients. Our results revealed that lower concentrations of CRP are related with improvement of chest-X-ray, suggesting the fine control of inflammatory response in these patients. Understanding the inflammatory mechanisms associated with treatment outcomes in pulmonary TB may lead to new therapeutic proposals to diminish the tuberculosis disease burden.

## Abbreviations

AFB, acid-fast bacilli; ATT, anti-tuberculous treatment; CRP, C-reactive protein; ESR, erythrocyte sedimentation rate; IGRA, Interferon gamma release assay; IQR, interquartile range; LP, left profile; MMP-8, matrix metalloproteinase-8; PA, posterior-anterior; PCA, principal component analysis; PTB, pulmonary tuberculosis; RR, relative risk

## Additional file

Additional file 1: Table S1. Spearman correlation values of the network analyses. (DOCX 17 kb)
