# Supplementary material for: Associations between systemic inflammation, mycobacterial loads in sputum and radiological improvement after treatment initiation in pulmonary TB patients from Brazil: a prospective cohort study
Source: BMC Infect Dis. 2016 Aug 5;16:368. doi: 10.1186/s12879-016-1736-3 (PMC4974760; doi:10.1186/s12879-016-1736-3)
Supplement: Additional file 1: Table S1. — Spearman correlation values of the network analyses. (DOCX 17 kb) [file 12879_2016_1736_MOESM1_ESM.docx]

| **Time point** | **variable 1** | **variable 2** | **r-value** | **p-value** |
| --- | --- | --- | --- | --- |
| pre-ATT | IFNg | IL4 | 0.3642 | 0.0177 |
|  | IFNg | TNFa | 0.729 | <0.0001 |
|  | IFNg | IL2 | 0.5672 | <0.0001 |
|  | IL10 | IL4 | 0.5499 | 0.0002 |
|  | IL10 | IL2 | 0.5144 | 0.0005 |
|  | IL4 | IL2 | 0.6263 | <0.0001 |
|  | IL6 | CRP | 0.4375 | 0.0038 |
|  | TNFa | IL10 | 0.5353 | 0.0002 |
|  | TNFa | IL6 | 0.3073 | 0.0477 |
|  | TNFa | IL2 | 0.7639 | <0.0001 |
|  | TNFa | IL4 | 0.6173 | <0.0001 |
| day 60 | CRP | ESR | 0.483 | 0.0002 |
|  | IFNg | IL10 | 0.6098 | <0.0001 |
|  | IFNg | IL6 | 0.5778 | <0.0001 |
|  | IFNg | IL4 | 0.4064 | 0.0068 |
|  | IFNg | CRP | 0.3934 | 0.0091 |
|  | IFNg | ESR | 0.3862 | 0.0105 |
|  | IFNg | IL2 | 0.3594 | 0.0179 |
|  | IL10 | IL4 | 0.5821 | <0.0001 |
|  | IL10 | IL2 | 0.5624 | <0.0001 |
|  | IL4 | IL2 | 0.8339 | <0.0001 |
|  | IL6 | CRP | 0.3894 | 0.0108 |
|  | TNFa | IL10 | 0.6639 | <0.0001 |
|  | TNFa | IL4 | 0.5943 | <0.0001 |
|  | TNFa | IL2 | 0.5256 | 0.0003 |

**Supplemental Table 1.** Spearman correlation values of the network analyses.
